# Special Issue “Transcription Factors in Plant Gene Expression Regulation, 2nd Edition”

**DOI:** 10.3390/ijms27114807

**Published:** 2026-05-27

**Authors:** Piotr Szymczyk

**Affiliations:** Department of Biology and Pharmaceutical Botany, Medical University of Łódź, Muszyńskiego 1, 90-151 Łódz, Poland; piotr.szymczyk@umed.lodz.pl

The second edition of the Special Issue entitled “Transcription Factors in Plant Gene Expression Regulation” contains five research and three review articles.

Among them, two research articles are dedicated to families of plant trans-factors (TFs) (Contributions 1,2). Qian et al. (2025) characterized five phosphate starvation response 1 genes in lettuce (*Lactuca sativa*) to reveal their responsiveness not only to phosphorus deficiency but also to darkness and phytohormone treatment (ABA, IAA, MeJA) (Contribution 1). Moreover, the Authors produced *LsaPHR1.1* knock-out mutants via CRISPR/Cas9-mediated genome editing to observe their improved biochemical parameters in comparison to wild plants under drought-induced stress conditions. *LsaPHR1.1* knock-out mutants indicated increased nitrate and ammonium contents, as well as elevated antioxidant and chlorophyll concentrations. Putatively, the *LsaPHR1.1* or related genes could be used in the future to improve phosphorus stress tolerance, resulting in better crop yield and quality (Contribution 1) [1,2].

The article of Li et al. (2026) analyses eighty-nine non-redundant basic helix-loop-helix (bHLH) TFs in *Rosa roxburghii* (*RrbHLHs*) (Contribution 2). The Authors observed their uneven distribution among seven chromosomes and classified them into twenty-three subfamilies. Seven *Arabidopsis* subfamilies were absent, suggesting a lineage-specific evolutionary route. The conserved motif and gene structure analyses indicated similarity of members within the same subfamily. However, the occurrence of subfamily specific variations suggested potential functional diversification. The Authors identified seventeen segmental and four tandem duplicated pairs, mainly evolving under purifying selection (Contribution 2). Closer examination of the presented pairs revealed their functional divergence during evolution, resulting from segmental duplication, combined with structural variations such as exonization/pseudoexonization, exon indels, and dissolution/joining. Analysis of promoter sequences indicated the presence of numerous *cis*-acting elements, related to tissue-specific regulation and phytohormone or stress response. Transcriptomic studies revealed that the expression of selected *RrbHLHs*, differentially responding to GA3 treatment, showed dynamic changes during *R. roxburghii* fruit development (Contribution 2).

Research by Zhang et al. (2026) concentrates on the understanding of functions of maize (*Zea mays*) bHLH TF, known as *ZmbHLH81*, in plant adaptation to drought stress (Contribution 3). Overexpression of *ZmbHLH81* in Arabidopsis enhanced drought tolerance, while CRISPR/Cas9-mediated mutagenesis in maize strongly increased plant responsiveness to drought stress, reflected in physiological changes such as accelerated water loss or increased malondialdehyde (MDA) concentration (Contribution 3). Combining the DNA affinity purification sequencing (DAP-seq) and transcriptomic studies enabled the identification of key downstream genes of ZmbHLH81. Among targets of ZmbHLH81 are the core ABA signaling kinase gene *ZmSnRK2.9* and stress-responsive TF genes *ZmNAC20* and *ZmHDZ4,* suggesting direct activation of a specific regulatory module, maintaining the ABA-mediated stomatal closure and reactive oxygen species (ROS) scavenging (Contribution 3) [3,4].

Regulatory networks of TFs participating in flower and fruit development or biosynthesis of bioactive compounds, such as diterpenoids, sugars, flavonoids, and anthocyanins, were studied in two research articles presented by Virág et al. (2025) and Zeng et al. (2026) (Contributions 4,5).

Virág et al. (2025) applied bioinformatic tools and databases to find cis-active motifs within promoters of eighteen flowering-related genes (Contribution 4). The Authors refined current models of floral development regulatory networks, providing valuable hypotheses related to cross-regulatory and autoregulatory circuits [5,6,7,8,9,10,11]. In total, thirty-six TFs binding to each of the eighteen promoters were identified as putative master regulators. Analysis of transcriptomic results after treatment with β-aminobutyric acid (BABA), delaying the flowering process, resulted in repression of *SQUAMOSA* and increased *DOF*-type TFs expression, which suggests chromatin-associated regulation of flowering activator suppression (Contribution 4).

Zeng et al. (2026) integrated Assay for Transposase-Accessible Chromatin sequencing (ATAC-seq) and RNA-sequencing results to analyze genome-wide chromatin accessibility and identify putative TFs involved in fruit development in the mulberry (*Morus atropurpurea* Roxb) variety Da10 (Yueshenda10) (Contribution 5). Motif enrichment analysis, integrated with the transcriptional regulatory network, provided a robust framework for the identification of candidate genes involved in the accumulation of key metabolites such as flavonoids, diterpenoids, and anthocyanins within the fruit maturation process (Contribution 5).

Three review articles present issues related to the role of bHLHs in the growth of cereal crops, CRABS CLAW in floral organ development and TFs maintaining functions of non-glandular and glandular secretory trichomes (Contributions 6–8).
